# A novel East African monopartite begomovirus-betasatellite complex that infects *Vernonia amygdalina*

**DOI:** 10.1007/s00705-016-3175-2

**Published:** 2016-11-29

**Authors:** Happyness G. Mollel, Joseph Ndunguru, Peter Sseruwagi, Titus Alicai, John Colvin, Jesús Navas-Castillo, Elvira Fiallo-Olivé

**Affiliations:** 1 Instituto de Hortofruticultura Subtropical y Mediterránea “La Mayora”, Universidad de Málaga - Consejo Superior de Investigaciones Científicas (IHSM-UMA-CSIC), Estación Experimental “La Mayora”, 29750 Algarrobo-Costa, Málaga Spain; 2 Mikocheni Agricultural Research Institute, P.O. Box 6226, Dar es Salaam, Tanzania; 3 National Crops Resources Research Institute, Namulonge, P.O. Box 7084, Kampala, Uganda; 4 Natural Resources Institute, University of Greenwich, Kent, ME4 4TB UK

## Abstract

The complete genomes of a monopartite begomovirus (genus *Begomovirus,* family *Geminiviridae*) and an associated betasatellite found infecting *Vernonia amygdalina* Delile (family Compositae) in Uganda were cloned and sequenced. Begomoviruses isolated from two samples showed the highest nucleotide sequence identity (73.1% and 73.2%) to an isolate of the monopartite begomovirus tomato leaf curl Vietnam virus, and betasatellites from the same samples exhibited the highest nucleotide sequence identity (67.1% and 68.2%) to vernonia yellow vein Fujian betasatellite. Following the current taxonomic criteria for begomovirus species demarcation, the isolates sequenced here represent a novel begomovirus species. Based on symptoms observed in the field, we propose the name vernonia crinkle virus (VeCrV) for this novel begomovirus and vernonia crinkle betasatellite (VeCrB) for the associated betasatellite. This is the first report of a monopartite begomovirus-betasatellite complex from Uganda.

**Electronic supplementary material:**

The online version of this article (doi:10.1007/s00705-016-3175-2) contains supplementary material, which is available to authorized users.

The genus *Begomovirus* is the largest of the seven genera in the plant virus family *Geminiviridae* [3, 19]. Begomoviruses are transmitted by the whitefly *Bemisia tabaci* (Hemiptera: Aleyrodidae) to a large variety of cultivated and wild-plant species [16]. Begomoviruses have a circular, single-stranded DNA genome, monopartite or bipartite, encapsidated in twinned icosahedral particles. Bipartite begomoviruses have two genome components, referred to as DNA-A and DNA-B, of similar size (2.5-2.8 kb), while monopartite begomoviruses have only one component, which is similar to DNA-A of bipartite begomoviruses. The DNA-A virion-sense strand encodes coat (CP) and pre-coat (pre-CP) proteins, the latter of which is present only in Old World (OW) begomoviruses. The DNA-A complementary-sense strand encodes the replication-associated protein (Rep), a transcriptional activator protein (TrAP), a replication enhancer protein (REn) and C4 protein. DNA-B encodes a nuclear shuttle protein (NSP) on the virion-sense strand and a movement protein (MP) on the complementary-sense strand. There are more than 300 accepted begomovirus species according to the recently updated demarcation criteria for the genus, which consider a DNA-A pairwise identity of 91% as the species threshold [4]. Recombination is a phenomenon that is crucial for speciation and evolution in the family *Geminiviridae* and contributes to the richness in species of the genus *Begomovirus.* This stresses the importance of recombination studies when analysing new begomoviruses.

Several types of DNA satellites have been described to be associated with begomoviruses: betasatellites [1], alphasatellites [2] and deltasatellites [11]. Betasatellites are circular, single-stranded DNA molecules about half the size of the begomovirus genome components that have been described to be associated with OW monopartite begomoviruses and are essential for induction of typical disease symptoms [1]. Betasatellite genomes contain an open reading frame in the complementary-sense strand encoding the βC1 protein, an A-rich region, a conserved stem-loop and a satellite conserved region.

*Vernonia amygdalina* Delile (family Compositae), known as bitter leaf, is a wild shrub that grows in tropical Africa and used in traditional medicine to treat malaria [13]. In this paper, we report the molecular characterization of a new monopartite begomovirus and associated betasatellite isolated from *V. amygdalina* plants from Uganda.

Samples from *V. amygdalina* plants showing crinkled leaves were collected in March 2015 from two locations in Uganda (sample UG7 from Naama [00°24.691′ N, 31°59.927′ E] and UG9 from Kawungera [00°27.761′ N, 31°39.171′ E]) (Fig. 1). Morphological identification of the plant samples was confirmed molecularly by DNA barcoding using chloroplast *rbcL* and *matK* genes [8]. Total DNA was extracted from leaf tissue using a modified CTAB method [17] and used as a template for rolling-circle amplification (RCA) using φ29 DNA polymerase (TempliPhi kit, GE Healthcare**)**. Amplified RCA products were digested with a set of restriction enzymes (*Bam*HI, *Eco*RI, *Hind*III, *Nco*I, *Nhe*I and *Sal*I), and both samples generated similar restriction patterns, which suggested the presence of a putative monopartite begomovirus (~2.8 kbp) and a putative DNA satellite (~1.3 kbp) in each sample. PCR was carried out using degenerate primers for DNA-B [18] but none of the samples yielded amplification products. Fragments of RCA products digested with *Eco*RI (~2.8 kbp) and *Nco*I (~1.3 kbp) were cloned into pBlueScript II SK (+) (Stratagene) and pGEM-T Easy Vector (Promega), respectively. Recombinant plasmid DNAs were introduced into *Escherichia coli* DH5α by electroporation, and selected clones were sequenced at Macrogen Inc. (Seoul, South Korea). Initial sequence similarity comparison was performed using the BLAST program (http://www.ncbi.nih.gov/). Sequence alignments were performed using MUSCLE [5], pairwise identity scores were calculated using SDT (Sequence demarcation tool) [15], and MEGA 7 was used for phylogenetic analysis [9].

**Fig. 1:**
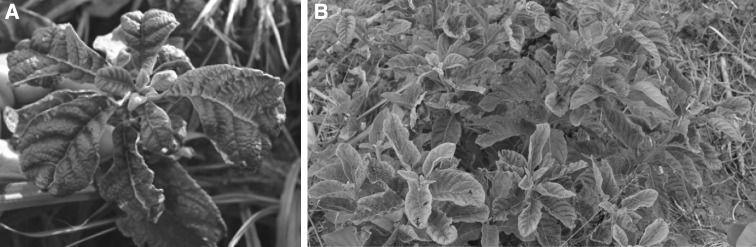
*Vernonia amygdalina* plants analyzed in this work showing crinkle symptoms on leaves (A, sample UG7; B, sample UG9)

Cloned ~2.8-kbp DNA from samples UG7 (2791 nt, KX831132) and UG9 (2791 nt, KX831133) had a genome organization typical of OW monopartite begomoviruses, with CP and pre-CP proteins encoded in the virion-sense strand, and Rep, TrAP, Ren and C4 proteins encoded in the complementary-sense strand. Begomoviruses from samples UG7 and UG9 showed the highest nucleotide sequence identity (73.1% and 73.2%, respectively) to an isolate of the monopartite begomovirus tomato leaf curl Vietnam virus (EU189150). DNA of ~1.3 kb cloned from the same samples (UG7, 1365 nt, KX831134; UG9, 1364 nt, KX831135) showed the typical genome organization of betasatellites (A-rich region, stem-loop, satellite conserved region and βC1 gene). Betasatellites from UG7 and UG9 showed the highest nucleotide sequence identity (67.1% and 68.2%, respectively) to vernonia yellow vein Fujian betasatellite (JF733779) found in *Cyanthillium cinereum* (L.) H.Rob. (syn. *Vernonia cinerea*) in China. The DNA-A-like genomes and betasatellites isolated from samples UG7 and UG9 were 99.4% and 97% identical, respectively. According to the begomovirus species demarcation threshold of 91% [4], the monopartite begomovirus reported here should be considered to belong to a new species. We propose that it be named vernonia crinkle virus (VeCrV) and that the two isolates be designated [Uganda-Naama UG7-2015] and [Uganda-Kawungera UG9-2015]. According to the recently proposed betasatellite species demarcation threshold of 91% (https://talk.ictvonline.org/files/proposals/taxonomy_proposals_plant1/m/plant02/6357), the betasatellite found in the same samples would represent a novel betasatellite, for which we propose name, vernonia crinkle betasatellite (VeCrB), and we suggest that the two isolates be designated [Uganda-Naama UG7-2015] and [Uganda-Kawungera UG9-2015].

Recombination is commonly detected in begomovirus and betasatellite genomes [6, 10, 14]. To detect putative recombinant fragments in the novel genomes, a search for potential parental begomoviruses and betasatellites in the GenBank database was conducted using SWeBLAST [7] with a window size of 200 and a step size of 200. The sequences with the highest SWeBLAST scores were selected for alignment using MUSCLE [5] and subsequent recombination analysis using the RDP4 package with default settings [12]. This analysis showed the presence of recombinant fragments in both VeCrV and the associated VeCrB (Supplementary Table S1). Interestingly, recombination events detected in VeCrV involve genomes from Asia and Africa.

Phylogenetic analysis showed that VeCrV isolates clustered with two OW begomoviruses from Africa, tobacco leaf curl Zimbabwe virus (AF350330) and tobacco leaf curl Comoros virus (AM701760) (Fig. 2A). In contrast, both VeCrB isolates grouped with betasatellites from Asia (vernonia yellow vein Fujian betasatellite [JF733779] and vernonia yellow vein betasatellite [FN435836]) (Fig. 2B). As additional begomovirus and betasatellite sequences from East Africa are discovered, this will enable more precise phylogenetic relationships of this new begomovirus and associated betasatellite to be determined.

**Fig. 2:**
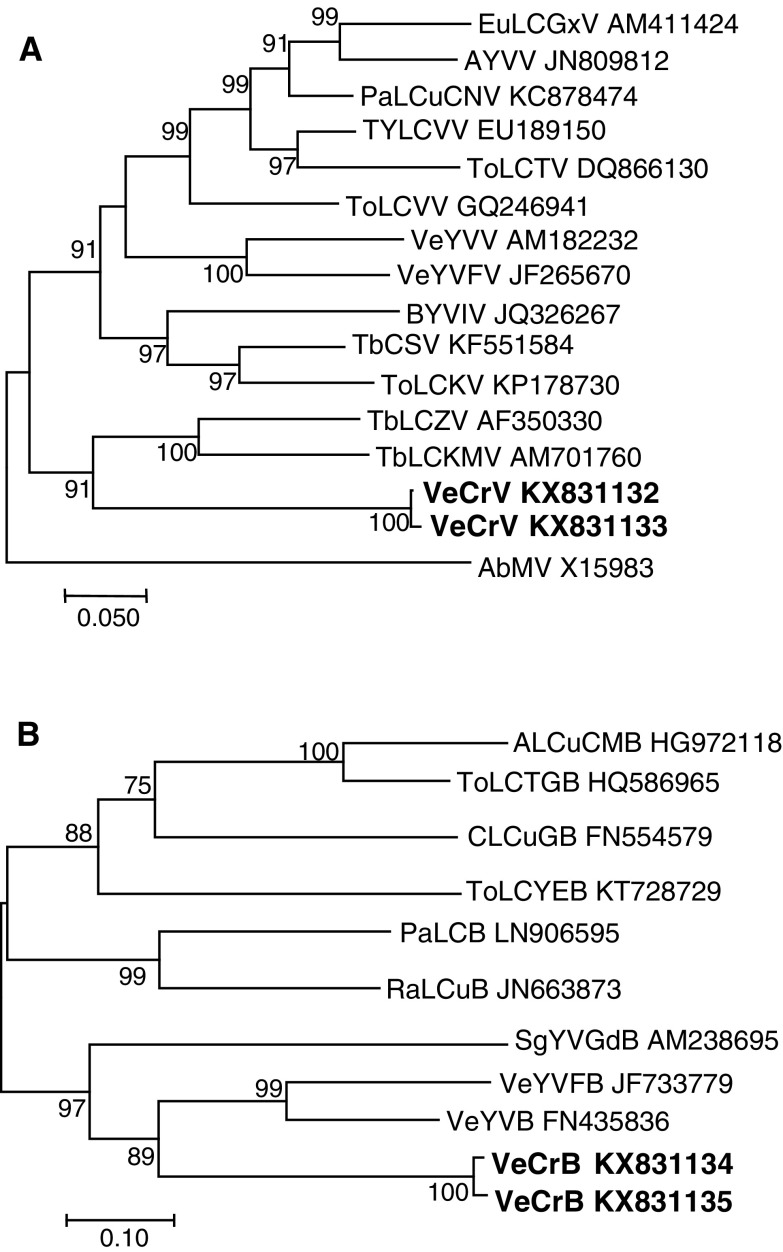
Phylogenetic tree showing the relationships between vernonia crinkle virus and other monopartite Old World begomoviruses (A) and vernonia crinkle betasatellite and other betasatellites (B). The trees were constructed by the maximum-likelihood method using the MEGA 7 program. Only bootstrap values >75% (1000 replicates) are shown. Viruses used to generate the phylogenetic tree (A) are as follows: AYVV, ageratum yellow vein virus; BYVIV, bhendi yellow vein India virus; EuLCGxV, euphorbia leaf curl Guangxi virus; PaLCuCNV, papaya leaf curl China virus; TbCSV, tobacco curly shoot virus; TbLCKMV, tobacco leaf curl Comoros virus; ToLCKV, tomato leaf curl Karnataka virus; TbLCZV, tobacco leaf curl Zimbabwe virus; ToLCTV, tomato leaf curl Taiwan virus; ToLCVV, tomato leaf curl Vietnam virus; TYLCVV, tomato yellow leaf curl Vietnam virus; VeYVFV, vernonia yellow vein Fujian virus; VeYVV, vernonia yellow vein virus. The DNA-A sequence of abutilon mosaic virus (AbMV), a New World begomovirus, was used as an outgroup. Betasatellite sequences used for the phylogenetic tree (B) are as follows: ALCuCMB, ageratum leaf curl Cameroon betasatellite; CLCuGB, cotton leaf curl Gezira betasatellite; PaLCuB, papaya leaf curl betasatellite; RaLCuB, radish leaf curl betasatellite; SgYVGdB, siegesbeckia yellow vein Guangxi betasatellite; ToLCTGB, tomato leaf curl Togo betasatellite; ToLCYEB, tomato leaf curl Yemen betasatellite; VeYVFB, vernonia yellow vein Fujian betasatellite; VeYVB, vernonia yellow vein betasatellite. The bar below each tree indicates nucleotide substitutions per site

This is the first report of a begomovirus-betasatellite complex infecting plants of the genus *Vernonia* in Africa and the first identification of a betasatellite in Uganda.

## Electronic supplementary material

Below is the link to the electronic supplementary material.

Supplementary material 1 (DOC 35 kb)
