# Supplementary material for: A novel East African monopartite begomovirus-betasatellite complex that infects Vernonia amygdalina
Source: Arch Virol. 2016 Nov 29;162(4):1079–82. doi: 10.1007/s00705-016-3175-2 (PMC5346420; doi:10.1007/s00705-016-3175-2)
Supplement: Supplementary file 1 — Supplementary material 1 (DOC 35 kb) [file 705_2016_3175_MOESM1_ESM.doc]

**Supplementary Table 1** Recombination events detected within the DNA sequences of VeCrV and VeCrB by at least five methods included in the RDP3 package

| **Recombinant** | **Recombination breakpoints** | **Parent-like sequences** | | ***p* value** | **Detection method*** |
| --- | --- | --- | --- | --- | --- |
|  |  | **Major** | **Minor** |  |  |
| VeCrV | 305-471 | ChiLCINV (KT948070) | TbLCTHV (KT322140) | 7.032x10^-25^ | G, B, M, C, S |
| VeCrV | 564-857 | TYLCMLV (LM651403) | TbLCZV (AM701756) | 1.300x10^-13^ | R, G, B, M, C, S, 3S |
| VeCrV | 2128-2361 | EACMV (KJ888092) | TbLCTHV (KT322140) | 2.577x10^-18^ | R, G, B, M, C, S, 3S |
| VeCrV | 2464-2563 | CoYMV (KT454834) | ToLCLKV (AF274349) | 4.226x10^-07^ | R, G, B, C, S |
| VeCrB | 1022-1060 | SgYVGdB (AM238695) | VeYVB (FN435836) | 4.659x10^-05^ | R, G, B, M, C |

*The method with the lower *p* value obtained for each region is underlined.

R, RDP; G, GENCONV; B, BootScan; M, MaxChi; C, Chimaera; S, SiScan; 3S, 3Seq.

ChiLCINV, chilli leaf curl India virus; TbLCTHV, tobacco leaf curl Thailand virus; TYLCMLV, tomato yellow leaf curl Mali virus; TbLCZV, tobacco leaf curl Zimbabwe virus; EACMV, East African cassava mosaic virus; CoYMV, cotton yellow mosaic virus; ToLCLKV, tomato leaf curl Sri Lanka virus; SgYVGdB, Siegesbeckia yellow vein Guangxi betasatellite; VeYVB, Vernonia yellow vein betasatellite.
